# Transposon-mediated BAC transgenesis in zebrafish and mice

**DOI:** 10.1186/1471-2164-10-477

**Published:** 2009-10-16

**Authors:** Maximiliano L Suster, Kenta Sumiyama, Koichi Kawakami

**Affiliations:** 1 Division of Molecular and Developmental Biology, National Institute of Genetics, Mishima, Shizuoka 411-8540, Japan; 2 Division of Population Genetics, National Institute of Genetics, Mishima, Shizuoka 411-8540, Japan; 3 Department of Genetics, The Graduate University for Advanced Studies (SOKENDAI), 1111 Yata, Mishima, Shizuoka 411-8540, Japan

## Abstract

**Background:**

Bacterial artificial chromosomes (BACs) are among the most widely used tools for studies of gene regulation and function in model vertebrates, yet methods for predictable delivery of BAC transgenes to the genome are currently limited. This is because BAC transgenes are usually microinjected as naked DNA into fertilized eggs and are known to integrate as multi-copy concatamers in the genome. Although conventional methods for BAC transgenesis have been very fruitful, complementary methods for generating single copy BAC integrations would be desirable for many applications.

**Results:**

We took advantage of the precise cut-and-paste behavior of a natural transposon, *Tol2*, to develop a new method for BAC transgenesis. In this new method, the minimal sequences of the *Tol2 *transposon were used to deliver precisely single copies of a ~70 kb BAC transgene to the zebrafish and mouse genomes. We mapped the BAC insertion sites in the genome by standard PCR methods and confirmed transposase-mediated integrations.

**Conclusion:**

The *Tol2 *transposon has a surprisingly large cargo capacity that can be harnessed for BAC transgenesis. The precise delivery of single-copy BAC transgenes by *Tol2 *represents a useful complement to conventional BAC transgenesis, and could aid greatly in the production of transgenic fish and mice for genomics projects, especially those in which single-copy integrations are desired.

## Background

BAC transgenesis has many important applications in basic and applied biomedical research, from the analysis of regulatory elements controlling gene expression to creating animal models of human diseases [1-3]. Because BACs can hold genomic segments as large as 300 kb, they are invaluable for the analysis of distant cis-regulatory elements [4], rescue of mutant phenotypes and regulated expression of human disease-related genes in mice [2,3]. A number of BACs encoding fluorescent reporters have been created by 'recombineering' in bacteria to visualize specific tissues or cell types *in vivo *[4-6].

BAC transgenesis has been carried out by microinjection of naked DNA into the cytoplasm of fertilized eggs in zebrafish [4,5] or oocyte pronucleus in mice [2,3]. With these methods, integration of the BAC in the genome occurs at best in ~5-20% of the injected survivors in mice [3] and ~1-3% in zebrafish [5]. A recent extensive study showed that about half of BAC transgenic mice carried 1 to 5 copies of the injected BAC as concatamers at one genomic locus, and the rest carried more than 5 copies, and up to 48 copies, arranged in various orientations [7]. Concatameric transgenes may be associated with silencing, instability, and genetic lesions both inside and around the transgenes [8,9] that seriously limit important experimental applications [10]. For example, Cre-mediated recombination between two *loxP *sites [2] placed at defined positions within a BAC can be used to delete precisely a candidate regulatory element, or to remove a cassette for conditional expression, and such applications may be facilitated by single copy integrations.

To deliver single-copy BAC transgenes to the genome, several methods including retroviral transfection into embryonic stem (ES) cells [11], site-specific homologous recombination in ES cells [5,12] and intracytoplasmic sperm injection [13] have been developed. While these methods are useful, they involve laborious handling of ES cells or freeze-thawing of sperm, and are not suitable to model organisms without established ES cell lines. Thus, simpler and more versatile methods for BAC transgenesis are still desired.

One potentially versatile approach for single-copy BAC transgenesis is to introduce BACs into the genome via DNA-type transposons. However, to date, no transposon has been reported to have the cargo capacity to carry such large DNA inserts as a BAC clone. The medaka fish *Tol2 *element is a DNA-type transposon that is active in a wide variety of vertebrate animals [14]. It has been shown that 200 bp and 150 bp from the left and right ends of *Tol2*, respectively, are *cis*-sequences essential for transposition, and *Tol2 *constructs that carried ~10 kb DNA inserts between them can transpose efficiently via a reliable cut-and-paste mechanism [15]. Here we demonstrate that *Tol2 *can carry a BAC clone and describe the first application of any transposon to BAC transgenesis in two major model organisms, zebrafish (*Danio rerio*) and mice (*Mus musculus*).

## Results and discussion

**Figure 1:**
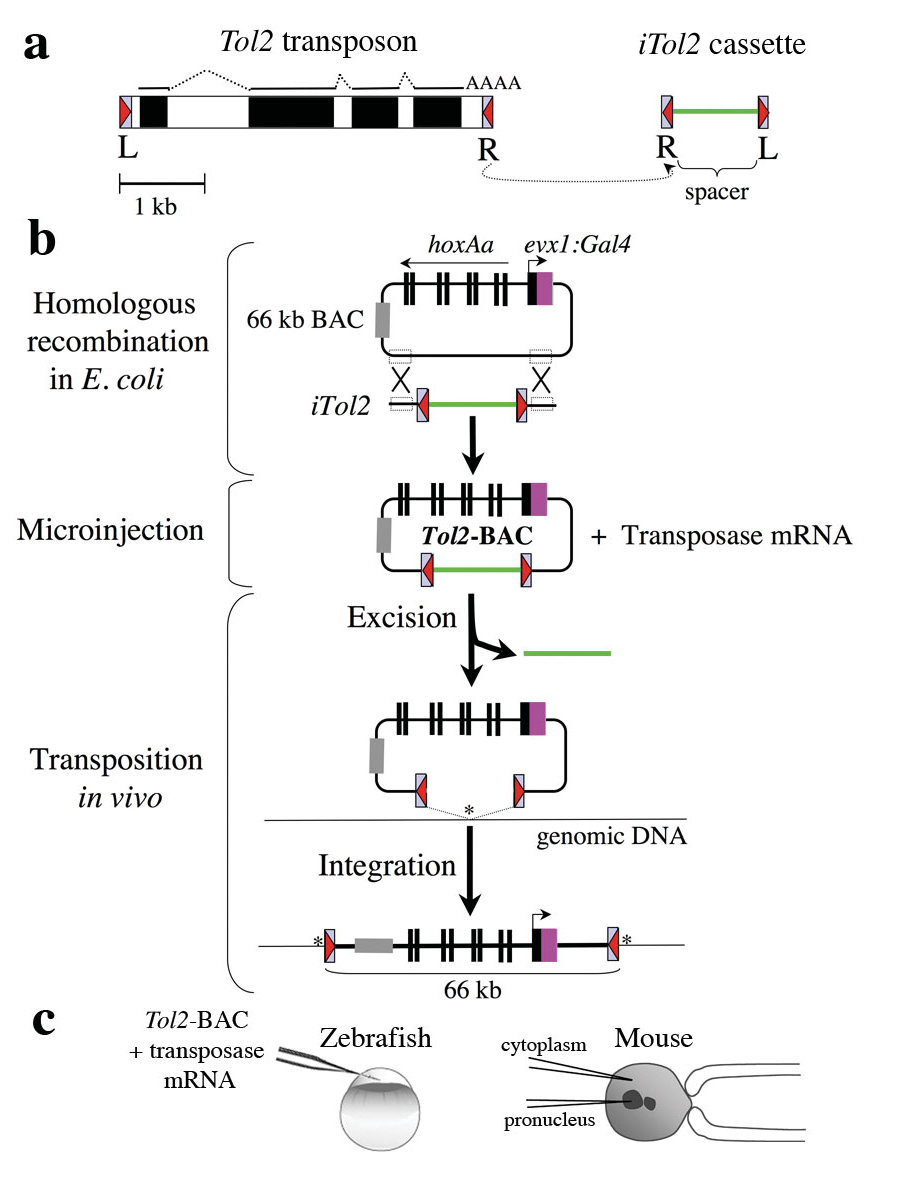
**A scheme for *Tol2*-mediated BAC transgenesis**. (a) Structures of *Tol2 *and *iTol2 *cassette. *Tol2 *encodes a single transposase mRNA (solid and dotted lines). The *cis*-sequences required for transposition, 200 bp from the left (L200) and 150 bp from the right (R150) are shown by red triangles. In the *iTol2 *cassette, these are inverted outwards. (b) The cassette was inserted into a Fugu *evx1 *BAC tagged with Gal4FF (purple box) that included part of the *hoxAa *cluster. The gray box indicates the pBeloBAC11 vector. The *Tol2*-BAC was co-injected with the transposase mRNA into zebrafish or mouse eggs. The *Tol2*-BAC portion was excised *in vivo *and integrates as a single copy into a single genomic locus (asterisk). (c) 50 ng/μl *Tol2*-BAC and 25 ng/μl transposase mRNA were co-injected into one-cell stage zebrafish embryos, or 14.5 ng/μl DNA and 10 ng/μl transposase mRNA into mouse oocytes (B6C3F1). A single needle was used to inject the DNA/RNA mixture into either pronucleus, cytoplasm or both of mouse oocytes.

To test whether *Tol2 *can carry a BAC, we used a BAC clone isolated from pufferfish (*Fugu rubripes*) that harbored ~59 kb genomic DNA containing the *hoxAa *cluster (*hoxa5 *through *hoxa13*) and the *evx1 *gene [16]. We inserted Gal4FF (a modified version of the yeast transcriptional activator Gal4) in frame with the second exon of *evx1 *by *galK*-mediated homologous recombination in *E. coli *[17]. It is expected that Gal4 directs GFP expression in the spinal neurons when the transgenic line is crossed with the UAS:GFP reporter fish [18]. To introduce the *Tol2 *sequence into the BAC plasmid, we designed a cassette containing the minimal *cis*-sequences of *Tol2 *in an inverted orientation separated by a ~1 kb spacer, which we termed *iTol2 *(Figure 1a and Methods). The *iTol2 *cassette enables incorporation of the *Tol2 *cis-sequences essential for transposition into a BAC clone through a single step of homologous recombination. The *Tol2*-*Frevx1*:*Gal4*-BAC plasmid (hereafter referred to as *Tol2*-BAC) contained 66 kb DNA, including the pufferfish genomic DNA and the backbone vector sequence, between the 200 bp and 150 bp end sequences of *Tol2 *(Figure 1b).

**Figure 2:**
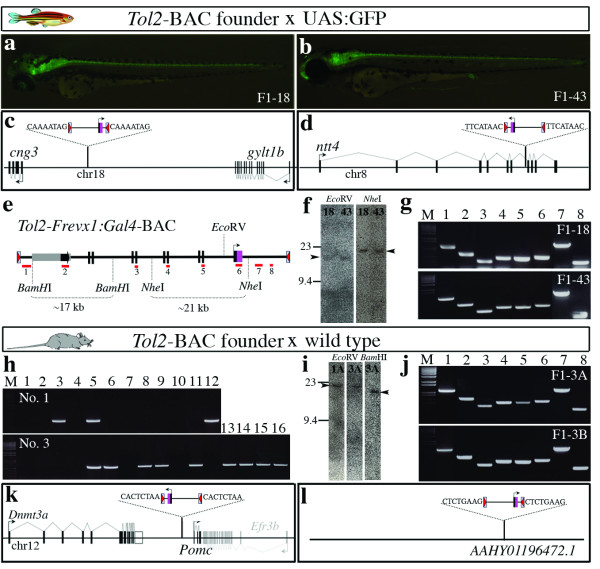
**Stable *Tol2*-BAC transgenesis in zebrafish and mice**. (a, b) Side views of double transgenic larvae (F1-18 and F1-43) carrying *Tol2*-*Frevx1*:*Gal4*-BAC (*Tol2*-BAC) and UAS:GFP that expressed GFP in spinal cord neurons at 2 dpf. (c) The insertion in F1-18 was located ~50 kb upstream of *cng3*. (d) The insertion in F1-43 was located in intron 7 of *ntt4*. 8 bp target site duplications are shown. (e) The structure of *Tol2*-BAC. *Tol2 cis*-sequences are shown by red triangles. Black bars indicate exons of *hoxa *and *evx1 *genes. A purple box indicates Gal4FF. The pBeloBAC11 vector is shown in gray and a black arrow within it is chloramphenicol. Positions of PCR fragments amplified (g, j) are shown as red lines with numbers. Positions of the *Bam*HI, *Nhe*I and *Eco*RV sites used for Southern blot are shown. (f) Southern blot analysis of transgenic fish (F1-18 and F1-43) by using a ^32^P-labeled Gal4FF probe. Single bands (arrowheads) were detected in DNA digested with *Eco*RV (~19-22 kb) and *Nhe*I (~21 kb). (g) Electrophoresis of PCR products amplified from transgenic fish. (h) PCR genotyping of F1 progeny from No.1 and No.3 founders mice with Gal4FF primers. (i) Southern blot analysis of F1 transgenic mice by using a ^32^P-labeled Gal4FF (left and middle panels) or Chloramphenicol (rightmost panel) probe. Single bands (arrowheads) were detected in DNA digested with *Eco*RV (~23 kb) or *Bam*HI (~20 kb). (j) Electrophoresis of PCR products amplified from transgenic mice. M, 1 kb ladder. (k) The F1-3A insertion was located near *Pomc*. (l) The F1-3B insertion was located in AAHY01196472.1.

We injected the *Tol2*-BAC plasmid and the transposase mRNA into the cytoplasm of zebrafish fertilized eggs. In 20 of 22 injected embryos, the ~66 kb DNA flanked by the *Tol2 cis*-sequences was excised from the donor plasmid, indicating that the transposase worked on the *iTol2 *cassette properly (Figure 1b, c; see additional file 1). Injected embryos were raised to sexual maturity and mated to homozygous UAS:GFP transgenic fish (Figure 2). We found that progeny from 2 of 50 injected fish showed GFP expression in the nervous system, including a medial population of spinal neurons, which is consistent with *Evx1 *expression in chicks and mice [16] (Figure 2a, b). Southern blot analysis confirmed the presence of single *Eco*RV bands (~20 kb) containing junction fragments of *Tol2 *and genomic DNA in these transgenic lines, indicating integration of single copies of the *Tol2*-BAC (Figure 2f). Inverse PCR identified that the *Tol2*-BAC was integrated 50 kb upstream of the *cng3 *gene on chromosome 18 (Figure 2c) or in the intron 7 of the *ntt4 *gene on chromosome 8 (Figure 2d) and created 8 bp duplications at the target site, indicating transposase-mediated integrations. PCR analysis using several sets of primers within the *Tol2*-BAC (Figure 2g and Methods) and Southern blot analysis using the internal *Nhe*I sites (Figure 2f) revealed that no gross structural alterations of the *Tol2*-BAC occurred upon integration.

Next, we tested whether *Tol2 *can carry a BAC in mice as well. We injected the *Tol2*-BAC plasmid into the pronucleus and cytoplasm or cytoplasm only of oocytes with the transposase mRNA (Figure 1c and see Additional file 2 - Table S1). As a control we injected the same DNA without transposase mRNA. 2 of 7 mice derived from pronuclear and cytoplasmic co-injection showed the presence of the *Tol2*-BAC DNA in the tail DNA as detected by PCR. In contrast, none of the mice derived from cytoplasmic injections (0 of 4) or control injections (0 of 7) had integrated the *Tol2*-BAC DNA (see additional file 3). The two PCR positive *Tol2*-BAC founder mice were raised to sexual maturity and crossed to wild type mice. We analyzed F1 offspring by PCR and found that 3 of 12 F1 from founder No.1 and 9 of 16 F1 from founder No.3 carried the *Tol2*-BAC transgene (Figure 2h). Southern blot analysis revealed that the progeny from founder No.1 and No.3 harbored single insertions at two different loci (1A and 3A, Figure 2i). We performed inverse PCR and cloned the *Tol2*-BAC genomic junction fragments (GenBank accession AB500928-AB500932). The 3A insertion from founder No.3 was located ~4.6 kb upstream of the *Pomc *gene on chromosome 12 (Figure 2k). In addition, another insertion (3B) was detected in the progeny of founder No.3 and mapped to contig AAHY01196472.1 (Figure 2k). 8 bp duplications were created at the integration sites, indicating that the integrations were mediated by transposition (Figure 2k, l). We did not examine founder No.1 further since the BAC integration site could not be mapped (data not shown). Further, we performed PCR analysis using several sets of primers within the *Tol2*-BAC and confirmed that no gross structural alterations of the *Tol2*-BAC occurred upon integration in 3A and 3B (Figure 2j). In addition, we used semi-quantitative PCR with BAC-specific primers to obtain an alternative estimate of BAC copy number. Consistent with our Southern blot and inverse PCR results, we estimated ~1-5 BAC copies in both zebrafish and mouse transgenics (additional file 4).

Our results demonstrate for the first time that a DNA transposable element has an extremely large cargo capacity sufficient for BAC transgenesis in two model vertebrates. *Tol2*-mediated BAC transgenesis should provide advantages over conventional DNA microinjection. First, it can generate single copy integrations that may be analyzed and mapped on the genome relatively easily. Second, such integrations may not suffer from problems that have been observed in concatemeric integrations of small linear DNA transgenes; i.e., gene silencing, gross rearrangements at the target loci, unwanted mutant phenotypes, etc. Third, it ensures integration of DNA from end to end without obvious rearrangements inside. All of these features have been observed in transgenesis using transposons with smaller inserts in mice and zebrafish [19,20]. The numbers of transposon insertions transmitted to F1 offspring were even lower in our BAC transgenic experiments probably because much fewer donor molecules were introduced in the nucleus during injection compared to small transposon constructs. While, to our knowledge, the 66 kb DNA is the largest insert for any transposon vector, we have also found that the transposase can catalyze transposition of *Tol2 *with a 120 kb BAC (additional file 5 and 6). Because *iTol2 *can be easily introduced into any existing BAC clone or BAC vector to create new BAC libraries, we are continuing further work to extend this method to constructs of larger sizes. While ongoing efforts are aimed at defining the optimal conditions and rate of *Tol2*-mediated BAC transgenesis, our current methodology should have immediate applications in comparative genomic analysis of cis-regulatory elements and gene functions in mice and zebrafish. Furthermore, it could contribute to development of transposon-based vectors for gene therapy, allowing precise delivery of a number of genes in the same context or of complete chromosomal loci in humans [1,10].

## Conclusion

Transposon-mediated BAC transgenesis is a new tool for precise delivery of BACs in both zebrafish and mice. It avoids potential multicopy concatameric integrations, allowing confirmation of the completeness of the BAC from end to end, as well as mapping of the genomic integration site. Therefore, transposon-mediated BAC transgenesis represents a useful complement to conventional BAC transgenesis, offering new possibilities in genomics research and beyond.

## Methods

### Construction of iTol2 cassette

PCR primers were designed to amplify the minimal ends of *Tol2 *[15] and connected in an inverted orientation with a ~1 kb DNA spacer.

### BAC recombineering

Fugu BAC 240G7 was obtained from Geneservice Ltd, UK and modified by *galK*-mediated recombination in bacteria [17]. Gal4FF was introduced in frame with exon 2 of *evx1 *[16]. The *iTol2 *cassette was introduced ~10 kb downstream of *evx1 *using primers:

F: cgcgaaataatgaaccctgctgcctcaggtatttatcacaaggcaatccgccctgctcgagccgggcccaagtg

R: ttcttctccaattcctgcaggttcggcggctgcggtctccagttcgagtcattatgatcctctagatcagatct

### Zebrafish transgenesis

50 ng/μl *Tol2*-BAC plasmid DNA and 25 ng/μl transposase mRNA were co-injected into one-cell stage zebrafish embryos (TL strain) according to established procedures [18]. Injected fish were raised to sexual maturity and outcrossed to identify transgenic carriers.

### Mouse transgenesis

*Tol2*-BAC plasmid DNA construct was dialyzed against buffer (5 mM Tris, 0.1 mM EDTA) and injected at 14.5 ng/μl with 10 ng/μl transposase mRNA into the pronucleus, cytoplasm or both of mouse oocytes (B6C3F1) according to standard procedures with minor modifications [2]. We carried out injection into pronucleus and cytoplasm by holding the injection needle for a second in cytoplasm after injecting into pronuclei; this way we deliberately permitted a certain amount of DNA solution to flow into the cytoplasm. We did not notice significant toxicity/death despite the relatively high concentration of the BAC DNA. We did not try lower concentrations of DNA because it was difficult to define a priori the 'most suitable' concentration of 'circular' BAC DNA. However, it has been reported that higher DNA concentrations enhance the overall integration rate for small plasmid constructs [21] and large BAC constructs [22] despite a lower survival rate. Injected embryos were transferred into the oviduct of the pseudopregnant female MCH mice. Newborn mice were genotyped by PCR using DNA extracted from the tails. The animal experiments were approved by the Animal Care and Use Committee of National Institute of Genetics.

### Inverse PCR

Zebrafish tail fin or newborn mouse limb genomic DNA was digested with *Alu*I or *Hae*III, followed by ligation and PCR according to published procedures with *Tol2*L-out: 5' ccctgctcgagccgggcccaagtg 3' and *Tol2*R-out: 5' attatgatcctctagatcagatct 3' and BAC-specific primers. Detailed protocols are available upon request.

### Genomic PCR

Internal fragments within *Tol2*-BAC in transgenic zebrafish or mice were amplified by PCR using the primers listed below. Lane No. refers to the numbers in Figure 2e and the respective PCR products shown in gels of Figure 2g and Figure 2j.

Lane No. Primer sequence (5' to 3')

1             F: ggagaagaaagaagcggcggcgatgcccgtcat

               R: cgccggccagtagtttacgacaggccattttaacg

2             F: atggagaaaaaaatcactggatataccaccg

               R: ttacgccccgccctgccactcatcgcag

3             F: atgtcgacatccggaacgctgactag

               R: ccggccatgggcagggaaccggac

4             F: atgatggattttgacgaaagggtcc

               R: gctctccgacgcgtttccgtacgcc

5             F: atgacaacgtcactgcttctccgtc

               R: gatagggccccgaagggtaggtcgg

6             F: atgaagctactgtcttctatcgaacaagcatgcg

               R: cgatctcgatatgctcccgggtaactaa

7             F: attcatcctcatcagacgcaccgcac

               R: gccacataggtacccctagatttgtt

8             F: ccccatcgcagggcagatcagaggtgag

               R: gccctctaaaagcctccatgtcgcc

## Abbreviations

BAC: Bacterial artificial chromosome.

## Authors' contributions

MLS designed the research, produced and analyzed most of the data and wrote the manuscript. KS performed mouse transgenesis and analyzed mouse data. KK designed the project, analyzed the data, and wrote the manuscript. All authors read and approved the final manuscript.

## Supplementary Material

Additional file 1 ***Tol2*-mediated BAC excision in early zebrafish embryos**. Transient *in vivo *assay of *Tol2*-mediated BAC excision in zebrafish embryos, including DNA microinjection procedure and demonstration of *iTol2 *cassette excision by PCR. Click here for file

Additional file 2 **Table S1. Survival and *Tol2*-mediated BAC integration efficiency upon microinjection in mice**. Number of surviving and PCR positive embryos for the transgene were counted after injection of BAC DNA with or without transposase mRNA in mouse oocytes. Click here for file

Additional file 3 **Minimal requirements for *Tol2*-mediated BAC transgenesis in mouse oocytes**. Schematic of *Tol2*-BAC microinjection into the mouse oocyte and PCR genotyping of founder mice injected with or without transposase mRNA into either cytoplasm or pronucleus or both. Click here for file

Additional file 4 **Estimation of *Tol2*-BAC copy number in transgenic zebrafish and mice**. Semi-quantitative PCR on BAC transgenic zebrafish and mouse genomic DNAs. PCR gel band intensities generated from transgenic DNAs were compared to those of control BAC DNA standards to produce an estimate of copy number. Click here for file

Additional file 5 ***Tol2*-mediated excision of a 120 kb BAC clone in zebrafish embryos**. Schematic of a 120 kb zebrafish BAC clone containing an *iTol2 *cassette and detection of its excision *in vivo *by PCR. Click here for file

Additional file 6 **Figure legends**. Detailed legends of additional files Click here for file
