# Supplementary figures and images for: Transposon-mediated BAC transgenesis in zebrafish and mice
Source: BMC Genomics. 2009 Oct 16;10:477. doi: 10.1186/1471-2164-10-477 (PMC2768751; doi:10.1186/1471-2164-10-477)

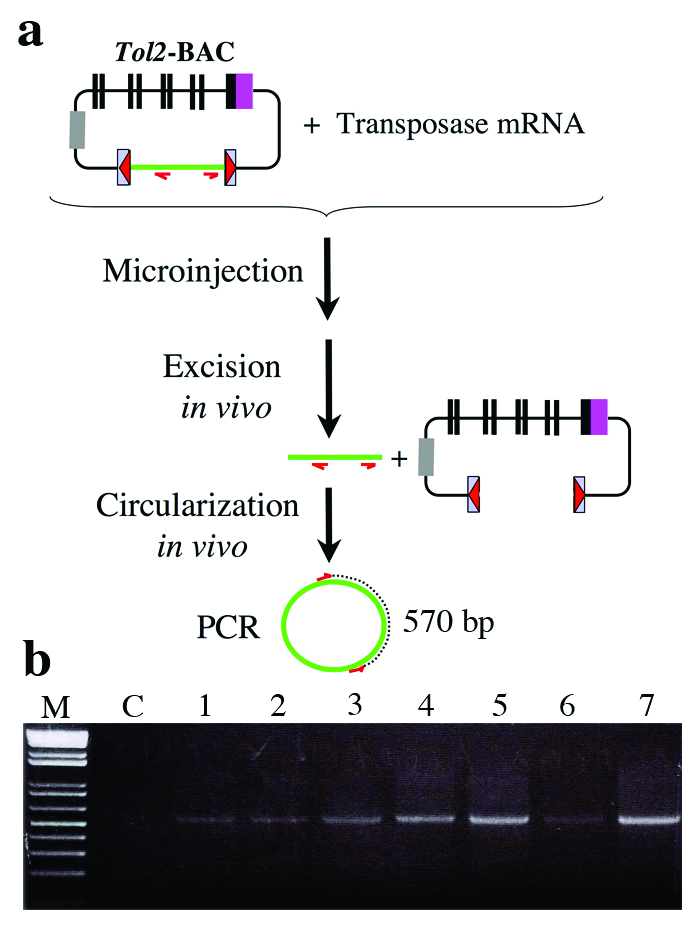

Supplement: Additional file 1 — Tol2-mediated BAC excision in early zebrafish embryos. Transient in vivo assay of Tol2-mediated BAC excision in zebrafish embryos, including DNA microinjection procedure and demonstration of iTol2 cassette excision by PCR. [file 1471-2164-10-477-S1.TIFF]

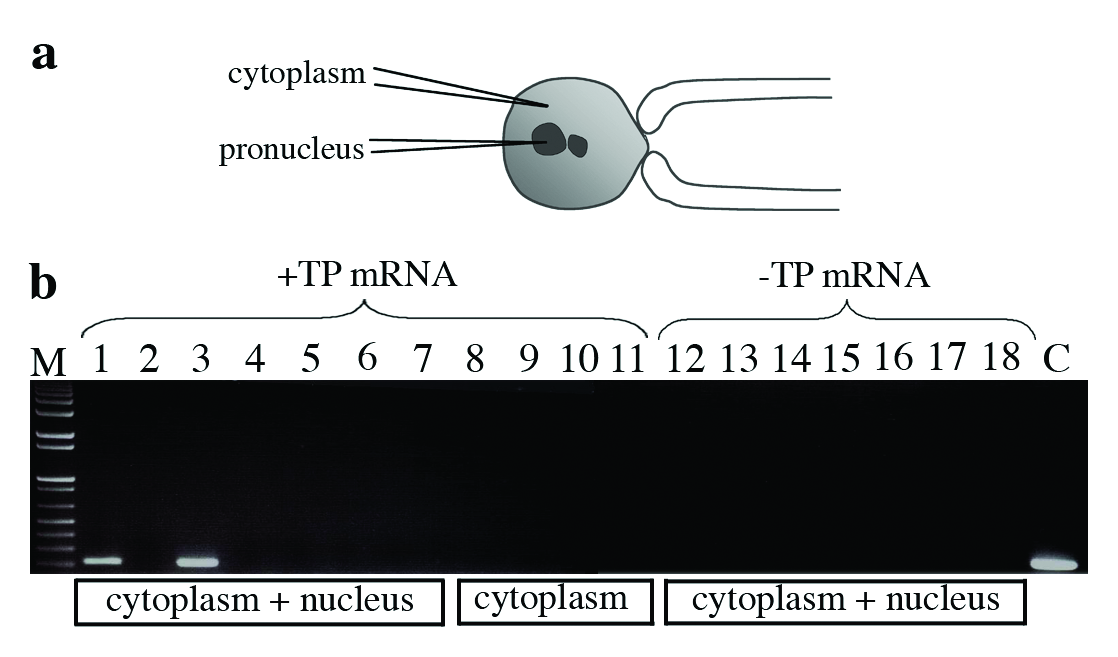

Supplement: Additional file 3 — Minimal requirements for Tol2-mediated BAC transgenesis in mouse oocytes. Schematic of Tol2-BAC microinjection into the mouse oocyte and PCR genotyping of founder mice injected with or without transposase mRNA into either cytoplasm or pronucleus or both. [file 1471-2164-10-477-S3.TIFF]

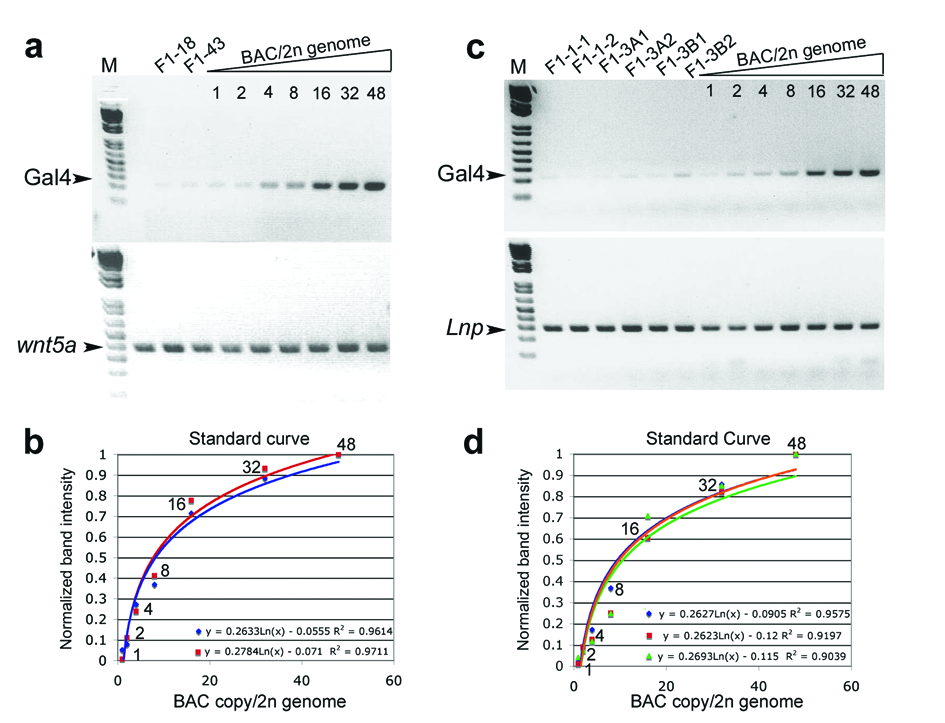

Supplement: Additional file 4 — Estimation of Tol2-BAC copy number in transgenic zebrafish and mice. Semi-quantitative PCR on BAC transgenic zebrafish and mouse genomic DNAs. PCR gel band intensities generated from transgenic DNAs were compared to those of control BAC DNA standards to produce an estimate of copy number. [file 1471-2164-10-477-S4.TIFF]

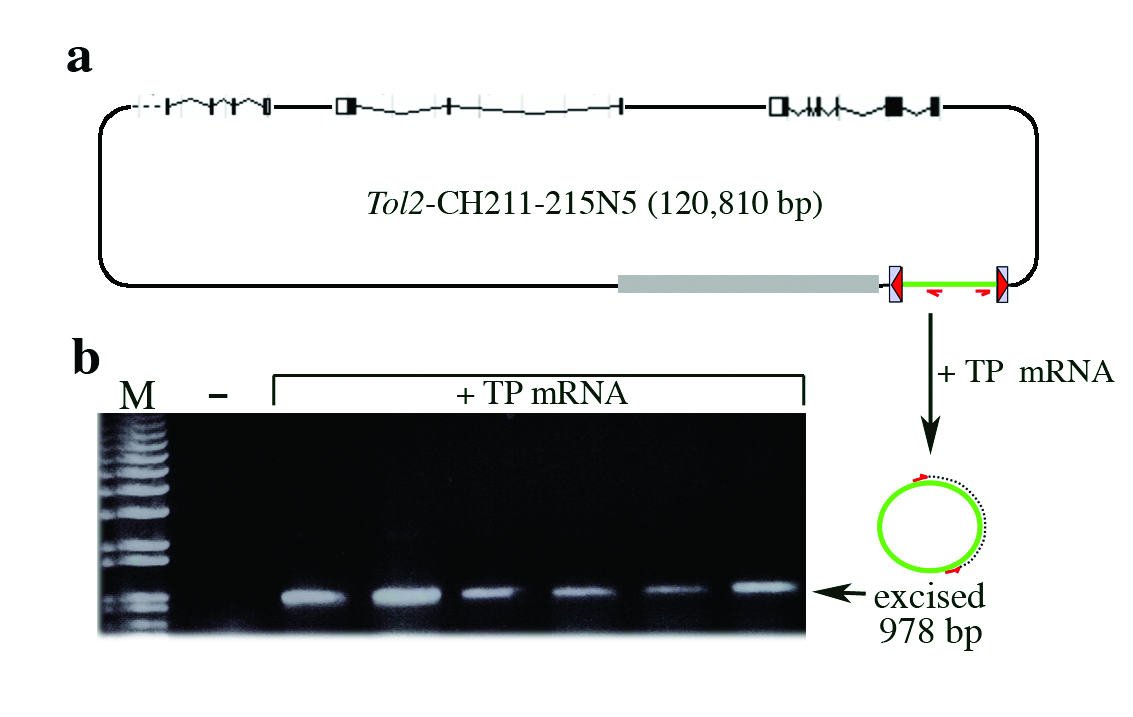

Supplement: Additional file 5 — Tol2-mediated excision of a 120 kb BAC clone in zebrafish embryos. Schematic of a 120 kb zebrafish BAC clone containing an iTol2 cassette and detection of its excision in vivo by PCR. [file 1471-2164-10-477-S5.TIFF]
