# Supplementary material for: Transposon-mediated BAC transgenesis in zebrafish and mice
Source: BMC Genomics. 2009 Oct 16;10:477. doi: 10.1186/1471-2164-10-477 (PMC2768751; doi:10.1186/1471-2164-10-477)
Supplement: Additional file 2 — Table S1. Survival and Tol2-mediated BAC integration efficiency upon microinjection in mice. Number of surviving and PCR positive embryos for the transgene were counted after injection of BAC DNA with or without transposase mRNA in mouse oocytes. [file 1471-2164-10-477-S2.DOC]

Additional File 5 – Table S1

| BAC Injection | Injection site | *n* injected | *n* transfer (%) | *n* born (%) | *n* PCR^+^ (%) |
| --- | --- | --- | --- | --- | --- |
| + TP mRNA | P + C | 113 | 92 (81.4%) | 7 (8%) | 2 (2%) |
| – TP mRNA | P + C | 194 | 166 (85.6%) | 7 (4.2%) | 0 (0%) |
| + TP mRNA | C | 19 | 13 (68.4%) | 4 (30%) | 0 (0%) |

P, pronucleus. C, cytoplasm. BAC DNA was 14.5 ng/μl and TP mRNA was 10 ng/μl.
