# Supplementary material for: Transposon-mediated BAC transgenesis in zebrafish and mice
Source: BMC Genomics. 2009 Oct 16;10:477. doi: 10.1186/1471-2164-10-477 (PMC2768751; doi:10.1186/1471-2164-10-477)
Supplement: Additional file 6 — Figure legends. Detailed legends of additional files [file 1471-2164-10-477-S6.DOC]

**Legends**

**Additional file 1**

(a) 50 ng/μl *Tol2*-BAC and 25 ng/μl transposase mRNA were co-injected into the blastomere of 1-cell stage zebrafish embryos. The BAC insert is excised *in vivo*, and *iTol2* linker (green) is re-ligated to form a circle. *iTol2* internal primers (red arrows) were used to amplify the excision product (570 bp) by PCR. (b) Electrophoresis of PCR excision products amplified from ~ 12 hr old embryo extracts. M, 1kb ladder. C, no extract. Excision PCR primers: 5’out: gcttgccgtaggtggcatcgccct, 3’out: gtgagtcgtattacgtagatccagac.

**Additional file 2**

(a) Schematic of the *Tol2*-BAC injection into the mouse oocyte. DNA with TP mRNA (+TP mRNA) or without (-TP mRNA) was injected into either cytoplasm or pronucleus or both. (b) PCR genotyping of injected founder mice. Gal4FF-specific primers were used to amplify a 250 bp fragment from tail genomic DNA, and separated on an agarose gel. M, 1 kb ladder. C, *Tol2*-BAC DNA.

**Additional file 3**

(a) Agarose gel electrophoresis of PCR amplicons from the BAC transgene (Gal4, top panel) or genomic internal control (*wnt5a*, bottom panel) from both transgenic (F1-18, F1-43) and BAC control standards (1-48). BAC copy standards were created by mixing known amounts of BAC DNA with control genomic DNA (30 ng zebrafish, 60 ng mouse) to create the equivalent of 1,2,4,8,16,32 and 48 copies per diploid (2n) genome. (b) Standard curve from two independent experiments. The mean grey value of each band was estimated using the measure tool in NIH ImageJ 1.37v and normalized to the 48x copy standard. Logarithmic regression curves were fitted to the data using y = *slope* x ln(x) – y_o_. (c) PCR amplicons from the BAC transgene (Gal4, top panel) or genomic internal control (*Lnp*, bottom panel) in mouse transgenics and BAC copy standards (1-48). (d) Standard curves were derived from quantification of band intensity as in b in 3 independent experiments. BAC copy numbers were estimated as 2e^(relative intensity –y intercept)/slope^. Copy number estimates were as follows: zebrafish F1-18 (1.7, 5.1), F1-43 (1.8, 3.9), mouse F1-1-1 (1.7 ± 0.2), F1-1-2 (1.4± 0.1), F1-3A1 (1.7 ± 0.1), F1-3A2 (1.6 ± 0.1), F1-3B1 (1.5 ± 0.02) and F1-3B2 (1.6 ± 0.2).

Semi qPCR primers:

Gal4FF: F: atgaagctactgtcttctatcgaacaagcatgcg, R: ttcagacacttggcgcacttcgg,

*wnt5a*: F: cagttctcacgtctgctacttgca, R: acttccggcgtgttggagaattc**,**

*Lnp*: F: gccccaagttgtgctttgctggatctgttac, R: gtgggctgggcccttttaatcaattggc

**Additional File 4**

(a) The *iTol2* cassette was introduced into the plasmid backbone of zebrafish BAC CH211-215N5 by homologous recombination in bacteria. The final Tol2-CH211-215N5-BAC plasmid was injected into the cytoplasm of 1-cell zebrafish embryos at 50 ng/μl together with 25 ng/μl of *Tol2* TP mRNA. (b) *In vivo* excision of the *iTol2* cassette linker (green) from the 120 kb BAC. A 978 bp excision product was detected by PCR only in embryos co-injected with both DNA and TP mRNA (+TP mRNA).

Excision PCR primers:

F, ggcgcacaaatcgcgcttaacggtc

R, cacggtttgataatcaatcgcgcag

**Additional File 5 – Table S1**

Analysis of survival rates and integration efficiency after injection of BAC DNA with (+) or without (-) transposase (TP) mRNA in mouse oocytes. The number (*n*) of embryos injected, transferred to surrogate mothers, born and PCR positive for the transgene were counted.
